# Clinical and Genetic Analysis of Multiple Osteochondromas in a Cohort of Argentine Patients

**DOI:** 10.3390/genes13112063

**Published:** 2022-11-07

**Authors:** Silvia Caino, Marisa Angelica Cubilla, Romina Alba, María Gabriela Obregón, Virginia Fano, Abel Gómez, Lorena Zecchini, Pablo Lapunzina, Miriam Aza-Carmona, Karen E. Heath, Carla Gabriela Asteggiano

**Affiliations:** 1 Servicio de Crecimiento y Desarrollo, Hospital de Pediatría J.P, Garrahan, Buenos Aires C1245, Argentina; 2 Centro de Estudio de las Metabolopatías Congénitas (CEMECO), Hospital de Niños de la Santísima Trinidad, Facultad de Ciencias Médicas, Universidad Nacional de Córdoba, Córdoba X5014AKN, Argentina; 3 Consejo Nacional de Investigaciones Científicas y Técnicas (CONICET), Córdoba X5000IND, Argentina; 4 Servicio de Genética, Hospital de Pediatría J.P. Garrahan, Buenos Aires C1245, Argentina; 5 Servicio de Ortopedia y Traumatología, Hospital de Niños de la Santísima Trinidad, Córdoba X5014AKN, Argentina; 6 Institute of Medical & Molecular Genetics (INGEMM), IdiPAZ, Hospital Universitario La Paz, 28046 Madrid, Spain; 7 ITHACA-European Reference Network (ERN-ITHACA), Hospital Universitario La Paz, 28046 Madrid, Spain; 8 CIBERER, ISCIII, 28029 Madrid, Spain; 9 Skeletal Dysplasia Multidisciplinary Unit (UMDE-ERN BOND), Hospital Universitario La Paz, 28046 Madrid, Spain; 10 Cátedra Farmacología, Carrera Medicina, Facultad de Ciencias de la Salud, Universidad Católica de Córdoba, Córdoba X5000IYG, Argentina

**Keywords:** osteochondroma, O-glycosylation disorders, multiple osteochondromatosis, multiple exostosis, EXT1/EXT2-CDG

## Abstract

Multiple Osteochondromatosis (MO, MIM 133700 & 133701), an autosomal dominant O-glycosylation disorder (EXT1/EXT2-CDG), can be associated with a reduction in skeletal growth, bony deformity, restricted joint motion, shortened stature and pathogenic variants in two tumor suppressor genes, *EXT1* and *EXT2.* In this work, we report a cross-sectional study including 35 index patients and 20 affected family members. Clinical phenotyping of all 55 affected cases was obtained, but genetic studies were performed only in 35 indexes. Of these, a total of 40% (*n* = 14) had a family history of MO. Clinical severity scores were class I in 34% (*n*:18), class II in 24.5% (*n*:13) and class III in 41.5% (*n*:22). Pathogenic variants were identified in 83% (29/35) probands. We detected 18 (62%) in *EXT1* and 11 (38%) in *EXT2*. Patients with *EXT1* variants showed a height z-score of 1.03 SD lower than those with *EXT2* variants and greater clinical severity (II–III vs. I). Interestingly, three patients showed intellectual impairment, two patients showed a dual diagnosis, one Turner Syndrome and one hypochondroplasia. This study improves knowledge of MO, reporting new pathogenic variants and forwarding the worldwide collaboration necessary to promote the inclusion of patients into future biologically based therapeutics.

## 1. Introduction

Congenital disorders of glycosylation (CDG) are a rapidly growing family of inherited metabolic defects comprising of >150 genetic diseases due to alterations in the N- or O-glycosylation pathway [1].

Multiple Osteochondromatosis (MO; MIM 133700, 133701), also known as EXT1/EXT2-CDG [2], is an autosomal dominant disease and the most frequent Congenital Disorder of O-Glycosylation (CDG) (1:20,000) [3]. Osteochondroma generally occurs as a single lesion, and most do not have a genetic component. When two or more osteochondromas are present, they are more likely to have a genetic cause [4,5,6,7]. Two tumor suppressor genes have been identified: *EXT1* (8q24.11-q24.13) and *EXT2* (11p12-p11), which encode two glycosyltransferases involved in the synthesis of heparan sulfate proteoglycans (HSPGs) [8,9,10,11,12,13,14,15]. The altered O-glycosylation of the heparan sulfate proteoglycans disturb binding of specific growth factors in chondrocytes, resulting in abnormal signaling and altered endochondral ossification leading to osteochondromas [16,17].

MO mainly affects the metaphysis of long bones or the surface of flat bones leading to bone tissue deformities. Osteochondromas can be associated with a reduction in skeletal growth, bony deformity, restricted joint motion, shortened stature, premature osteoarthritis and compression of peripheral nerves. The malignant transformation of osteochondroma to secondary peripheral chondrosarcoma has been reported in 0.5–5% of cases. Pain, acute ischemia and signs of phlebitis or nerve compression are associated with the most severe forms [5,6,18].

Heterozygous single nucleotide variants, deletions, or duplications resulting in frameshifts or loss of *EXT1* and *EXT2* expression are identified in approximately 80% of patients with MO. Structural alterations involving single or multiple exons of *EXT1* or *EXT2* have been found in an additional 10% of cases [5,6,19,20,21,22,23,24]. To date, more than 875 *EXT1* and 450 *EXT2* different pathogenic variants have been found worldwide (https://databases.lovd.nl/shared/genes/EXT2/EXT1, accessed on 1 August 2022) [25].

In the present study, we report the clinical studies and phenotypic data, together with the genetic variants, identified in a cohort of Argentine patients with MO, from a single center.

## 2. Cohort of Patients and Methods

This was an observational, cross-sectional study of a cohort of patients with clinical and radiological diagnosis of MO seen for the first time and/or during follow-up at the skeletal dysplasia clinic of Garrahan Hospital, Buenos Aires, Argentina. All patients older than two years of age and affected parents registered in the database were considered eligible. Those patients who could not be located, who did not attend the consultation and/or who did not agree to participate in the study were excluded. For the clinical analysis, three cases that presented additional genetic conditions were eliminated: Turner syndrome (*n*:1), Larsen syndrome (*n*:1) and Hypochondroplasia (*n*:1). Informed consent for participation in the study was obtained from the probands or parents. The study was approved by the Ethics Committee (CIEIS, Act No. 95/2007/2016).

### 2.1. Clinical Studies and Phenotypic Data

Clinical phenotyping from all 55 patients (35 indexes and 20 family affected members) was obtained. Each patient was evaluated by a multidisciplinary team and a complete physical examination was performed, including height measurement, pubertal development according to Tanner [26], and a pain survey. Height was measured following the recommendations of the Argentine Society of Pediatrics [26]. Gender, age at time of consultation, affected relatives, schooling and place of residence were also recorded. Radiographs of the entire skeleton were assessed. The adults considered were those patients with bone age and adult pubertal development. Other variables obtained by reviewing the clinical history, and validated by questioning, were the age of first symptom, number of previous surgeries and extra-skeletal complications, such as pneumothorax, hemothorax, compression of peripheral nerves, paresis, and malignancy of the injuries.

Clinical severity was defined following the classification of Pedrini et al. [27] with three groups based on the presence of deformities (shortening of long bones, curvature, scoliosis, varus or valgus of the knee, ankle deformity) and functional limitations. Group I: without deformities or functional limitations (A ≤ 5 sites with exostoses; B > 5 sites with exostoses); Group II: deformities without functional limitations (A ≤ 5 sites with deformity; B > 5 sites with deformity); and Group III: deformities and functional limitations (A functional limitation in one place; B in more than one site).To avoid an excessive dispersion of data in the statistical analysis, we elected not to consider the clinical subclassification (A and B) in each group.

### 2.2. Genetic Screening

Genomic DNA was obtained from peripheral blood leukocytes from all 35 index individuals using the Wizard Genomic DNA purification Kit (Promega, Madison, WI, USA), according to the manufacturer’s instructions. Genetic screening of the 11 *EXT1* (NM_000127.3) and 13 *EXT2* (NM_000401.3) coding exons and their intronic flanking regions were performed by either PCR/Sanger sequencing using primer sequences and PCR conditions as described by Delgado M.A. et al., 2014 [6] or by a skeletal dysplasia Next Generation Sequencing panel (SkeletalSeqV5, *n* = 368 genes, SeqCap EZ (Roche Nimblegen Inc., Foster, CA, USA) on a NextSeq sequencer (Illumina, Inc., Foster, CA, USA) [28]. Genetic studies were complemented by additional CNV analysis using the NGS panel, SNP arrays (Infinium CytoSNP-850K v1.2 BeadChip, Illumina [29] and/or MLPA EXT1/EXT2 (P215-B1) analysis performed in DNA samples of those with previous negative results, following the manufacturer’s instructions (MRC-Holland, Amsterdam, The Netherlands). Variant nomenclature was according to HGVS nomenclature (www.hgvs.org, accessed on 1 August 2022) and classified according to the recommendations of the American College of Genetics and Genomics [30,31].

The identified variants were assessed for amino acid conservation in silico pathogenicity prediction analysis: CADD V1.4 (http://cadd.gs.washington.edu/, accessed on 1 August 2022), SIFT (https://sift.bii.a-star.edu.sg/, accessed on 1 August 2022), Polyphen (http://genetics.bwh.harvard.edu/pph2/, accessed on 1 August 2022), MutationTaster ((http://www.mutationtaster.org/, accessed on 1 August 2022), various splicing programs available in Alamut V2.14 (Interactive Biosoftware, Ruan, France), and allelic frequencies in gnomAD (https://gnomad.broadinstitute.org/, accessed on 1 August 2022). NGS Copy number variant (CNV) analysis was performed using an in-house tool, LACONv (INGEMM, Madrid, Spain). Variant nomenclature was according to HGVS nomenclature (www.hgvs.org, accessed on 1 August 2022) and classified according to the recommendations of the American College of Genetics and Genomics [30,31].

### 2.3. Statistical Analysis

Descriptive statistics were performed using absolute and relative frequencies for categorical variables and mean or median for continuous variables, depending on the dispersion of the data: standard deviation (SD) or interquartile range (IQR), respectively. The height z score (Pz) was calculated using LSM-Growth with respect to the Argentine population. Short stature was defined as a z score less than −2 SD. The cohort was analyzed according to an exploratory t-test and to the detected EXT1 or EXT2 variants to analyze the difference between the continuous variables (age, number of surgeries, age first symptom, height z score) and chi square or Fisher’s Exact test for categorical variables (gender, child/adult, pain, ≥10 osteochondromas, surgery, severity). For the severity variable, we consider Grade I versus Grades II and III given the small number of cases. The α level 0.05 and R 4.1.0 was used. The data was dissociated according to the Personal Data Protection Law.

## 3. Results

### 3.1. Clinical and Phenotypic Studies

**Figure 1:**
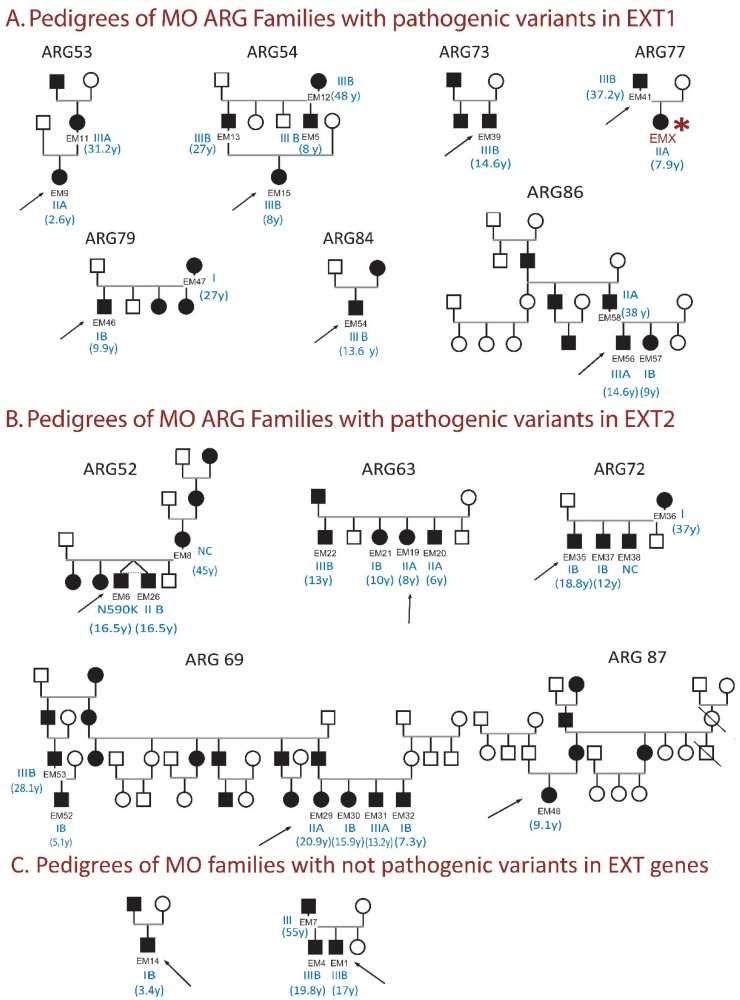
Segregation analysis in the ARG MO families. Indexes are marked with an arrow. Severity score and current age (in brackets) are shown in blue. Patients included in the clinical analysis were named with the prefix EM and a corresponding number. (**A**) Pedigrees of MO ARG Families with pathogenic variants in *EXT1*. The red asterisk in ARG77 (EM41) indicates that the patient was also included in the clinical analysis. (**B**) Pedigrees of MO ARG Families with pathogenic variants in *EXT2*. (**C**) Pedigrees of MO families with NO pathogenic variantes in *EXT1* or *EXT2*.

Clinical and radiological findings from 55 patients were obtained. From the indexes, 14 had a familiar inheritance and the 21 had sporadic mutations. Hence, only 14 families are described in Figure 1. Average age was 13.56 years old (r: 2.21–55.3); 63.6% (*n* = 35) were male and 36.4% (*n* = 20) female. Twenty-three cases had adult bone age. Forty-three cases lived in Buenos Aires and 12 in other provinces. The median age of the first symptom was 2.0 years (IQR 0.75–4.7) and no differences were found between familial forms and de novo variants. The most frequent locations of the first symptoms were knees and wrists. Main clinical features reported were chronic pain 74.5% (41/55), in 65.6% of children (21/32) and 87.0% of adults (20/23). Functional limitation in the upper limbs was observed in 21/51 (41.2%) cases and in the lower limbs in 14/51 (27.5%). The most frequent affectation is limitation in prono-supination in the upper limbs and in full flexion in the knees. Median z score of stature in children was −0.18 (IQR −0.93/0.7) SD and in adults −1.44 (IQR −2.43/−0.49) SD. Short stature was observed in 16.4% (9/55), 34.8% (8/23) of adults and 3.1% (1/32) of children (*p* = 0.002). Short stature was present in 4/35 (11.4%) of males and 5/20 (25%) of female (*p* = 0.19) (Table 1 [32,33,34,35,36,37,38,39,40,41,42,43,44,45,46,47,48,49]).

Sixty percent of patients (27/45) had 10 or more exostoses on radiographs and 40/50 (80%) brachy-metacarpals. Twenty-nine of 55 patients (52.7%) required surgery, with an average 1.7 (r: 1–11) surgeries per patient and a median age at first surgery of 6.2 years (r: 3.49–12.3). Functional alteration, limb axis deviation and pain were the reasons for which surgery was performed. A total of 11.4% (5/44) of patients presented spinal exostoses; one patient presented symptoms of spinal cord compression and required surgery. Leg length asymmetry greater than 1 cm was observed in 14.5% (8/55) of cases, with the average magnitude of length asymmetry being 2.2 cm (r: 1.5–4.0cm). We found intellectual disability and/or behavioral changes in 10.9% (6/55) of patients with normal brain image and karyotype, three of them with a family history of MO.

Clinical severity was classified according to the Pedrini score [27], class I in 34% (*n*:18), class II in 24.5% (*n*:13) and class III in 41.5% (*n*:22). Two patients could not be evaluated and classified. A total of 72.7% (16/22) of adults and 61.3% (19/31) of children presented a moderate-severe class of disease (p 0.38) (Table 1). Twenty three of 34 men and 12/19 women presented severity class II–III (p 0.74). A wide clinical variability was observed in most of the families, except family ARG54 (Figure 1) with a severe form in all the members studied and a family without variant detected (not included in Figure 1) with milder forms in both sexes.

### 3.2. Genetic Results

A total of 29 pathogenic variants in the 35 probands (29/35, 83%) were identified (Table 1). We detected: 18 variants (62%) in *EXT1* and 11 (38%) in *EXT2*; (35%) frameshifts, (35%) nonsense, (13%) splicing and (17%) missense. No *EXT1* or *EXT2* pathogenic variant was detected in 6 (17%) probands. However, one presented with a heterozygous variant of unknown significance (PM1, PM2, PP3) in *FLNB*, NM_001457.4:c.5908G>A p. (Glu1970Lys) (Larsen syndrome, MIM 150250). This variant affects a highly conserved amino acid in the filamin repeat domain. It is absent from gnomAD and has not been previously observed (HGMD Professional [31]). Two patients also had a dual diagnosis of a pathogenic *EXT2* variant, one of them presented X-chromosome monosomy (Turner syndrome) and another had an *FGFR3* pathogenic variant, NM_000142.4:c.1620C>A p. (Asn 540Lys) (Hypochondroplasia).

### 3.3. Genotype-Phenotype Correlations

Forty-eight cases, 27 index cases and 21 family members, were divided according to variants in *EXT1* or *EXT2* to explore clinical differences (Table 2). Cases EM6 and EM48, shown in Figure 1, were excluded from the analysis because they presented MO associated with hypochondroplasia and Turner syndrome, respectively. No significant differences for age, gender, family cases and child/adult ratio between the two groups were observed. The average height in both groups was less than the 50th centile. However, patients with *EXT1* variants showed a height z-score 1.03 SD lower than those with *EXT2* variants and greater clinical severity (II–III vs. I). Although the difference was not significant, the presence of pain, the number of exostoses, the cases that required surgery and the number of surgeries per patient were greater in the *EXT1* group (Table 2). Six patients with pathogenic variants in *EXT1* showed intellectual disability (low CI), normal brain image and karyotype.

Five index cases (and two family members) without a pathogenic variant detected in *EXT1* or *EXT2* with a median age of 16.8 years (IQR 6.8–22.0) showed a median height z score of −0.16 (IQR 0.11/−2.04). Four males (three from the same family and one from another family) presented clinical severity class III, with paternal inheritance, while three sporadic cases presented severity class I.

## 4. Discussion

In this study, the diagnosis of MO was first established from phenotypic characteristics and radiographic findings. The hereditary condition was observed in 40% of probands, which is similar to previous data in our country [6] but lower than the proportion of MO patients with family history reported in 36 worldwide cohorts (80%) The autosomal dominant condition characterized by heterozygous pathogenic variants in *EXT1* or *EXT2* were identified by a summatory of genetic tests. As described by other authors, *EXT1* variants were the most prevalent alterations [27,34,45]. No variant was detected in six (17%) probands, two familial and four sporadic, which is a similar percentage to some cohort studies [27,52] and higher than others [36]. Mosaic *EXT1/EXT2* variants have been reported in MO [53] and these can be detected using the NGS panel (unpublished data) but none were identified in this cohort. Thus, variants in unscreened regions of these genes (introns, etc.,) or in unidentified additional genes may explain these negative cases. One of these probands had a VUS in *FLNB* (Table 1). Two individuals had a dual diagnosis, with MO and Turner syndrome or MO and hypochondroplasia.

Nevertheless, the phenotype cannot be predicted based on variant type or which gene is mutated. In this study, we found lower height z-score and moderate-severe clinical severity by Pedrini’s classification, in cases with *EXT1* variants, findings previously observed in other populations [27,35]. In this sense, short stature was present in 35% of adults, similar to that described by other authors [54]. In contrast to that observed by Pedrini et al., we did not find differences in the percentage of short stature or severity between men and women [27].

A high prevalence of chronic pain was observed in both children and adults and was greater in patients with *EXT1* variants, although the difference was not significant. In previous studies, including one we performed [55], pain has a negative impact on quality of life [56]. There is a consensus about the patients’ treatment, related to the symptoms and complications. In this regard, functional alterations, pain and limb axis deviations were the reasons for surgery in our cohort. Although surgical procedures prior to the study could modify the clinical classification of severity, the number of patients who required surgery tended to be higher in cases with an *EXT1* variant, as well as the number of surgeries per child.

The molecular mechanism underlying the pathogenesis of MO is still unclear. Many studies have shown that the etiology of osteochondroma is largely due to genomic mutations in *EXT1* and *EXT2*, resulting in the loss or insufficient synthesis of glycosyltransferases which are related to HS synthesis [56]. Both genes encode glycosyltransferases, which are essential for the synthesis of HS, a polysaccharide present in all animal tissue cells and the extracellular matrix. HS covalently binds to core proteins to form heparin sulfate proteoglycans (HSPGs). HSPGs are localized in the cell membrane and extracellular matrix, which can bind to growth factors and participate in the signal transduction process of chondrocytes. These hetero-oligomeric complexes EXT1/EXT2 localized in the Golgi apparatus catalyze the HS synthesis process [57]. The truncated HSPG disturbs specific growth-factor binding in chondrocytes, resulting in abnormal signaling and altered endochondral ossification, thus leading to MO [9].

In this cohort, novel variants were identified, most of which were classified as pathogenic. The novel *EXT1* variants in the canonical splice sites, c.1164+2T>A and c.1284+1G>T (intron 3 and 4, respectively) and c.1173+1G>A (intron 7) rather than for *EXT2* showed a pathogenic effect that correlates with severity of the Grade III disease. A total of 83% of genomic variants are predicted to result in a truncated protein, 35% frameshifts, 35% nonsense variants and 13% splice variants. Missense variants were detected in only 17% of patients.

A high degree of genetic variability was observed due to the highly diverse ethnic origin of the Argentine population, as the consequence of the mixture of native genes with genes that come predominantly from European Mediterranean countries, especially Italy and Spain, and, to a lesser extent, from Central and Eastern Europe and the Middle East [58]. Indeed, some of the detected pathogenic variants have been previously reported in patients of Caucasian descent.

Six patients, with *EXT1* pathogenic variants, presented intellectual disability and/or behavioral problems. Three of them had no family history of osteochondromas. Recent studies report skeletal involvement together with other clinical manifestations including dysmorphism or multiple congenital anomalies and various degrees of developmental delay/intellectual disability including an *EXT1* MO patient [57]. Despite the estimated incidence of malignant degeneration to chondrosarcoma in 2–5% of patients [5,6] we did not observe cases, perhaps due to the young age of patients.

## 5. Conclusions

The implementation of NGS has substantially aided in the genetic diagnosis of MO. In this study, the clinical and genetic diagnoses were confirmed in 29/35 index patients presenting MO and many of them were novel variants. Initial diagnosis was by radiological findings, but interestingly, in our cohort, 40% of the probands had a family history of MO. Patients with *EXT1* variants showed a height z-score of 1.03 SD lower than those with *EXT2* variants and greater clinical severity (II–III vs. I). This study improves the diagnosis and knowledge of MO, reporting new pathogenic variants and forwarding the worldwide collaboration necessary to promote the inclusion of patients into future biologically based therapeutics.

## Figures and Tables

**Table 1:** Phenotypic data from MO index patients and pathogenic variants detected in *EXT1* and *EXT2* genes.

	Age (y)	Family History (FH)/Sporadic (S)	Sex (F/M)	Gene	Exon/ Intron	cDNA Variant	Predicted Protein	ACMG Classification	Height Z Score	Severity by PedriniScore *	PMID Reference
ARG53 (EM9)	2.6	FH	F	*EXT1*	Ex1	c.812A>G	p. (Tyr271Cys)	Likely pathogenic (PM2, PM5, PP3, PP4, PP5)	−0.8	IIA	[49] PMID: 24532482
ARG54 (EM15)	8.1	FH	F	*EXT1*	Ex10	c.1910dup	p. (Tyr637*)	Pathogenic (PVS1, PM2, PP4, PP5)	−1.8 neurological delay	IIIB	[42] PMID: 29529714
ARG55 (EM2)	9.1	S	M	*EXT1*	Ex6	c.1460T>C	p. (Val487Ala)	VUS (PM2, PP3, PP4, PM6, BP1)	−1.5	IIA	This study
ARG61 (EM3)	3.5	S	F	*EXT1*	Ex6	c.1432dup	p. (Ser478Phefs*43)	Pathogenic (PVS1, PM2, PP4, PP5)	1.6	IB	[43] PMID: 8981950 [44] PMID: 9521425 [11] PMID: 19810120 [37] PMID: 30334991
ARG62 (EM18)	10.2	S	M	*EXT1*	Int2	c.1056+1G>A	p. ?	Pathogenic (PVS1, PM2, PP4, PP5)	−1.9 neurological delay	IIIA	[39] PMID: 9150727 [48] PMID: 34409107
ARG64 (EM23)	20.4	S	M	*EXT1*	Ex5	c.1387G>T	p. (Gly463*)	Pathogenic (PVS1, PM2, PP4)	−0.6 neurological delay	IIIB	This study
ARG71 (EM34)	18.4	S	F	*EXT1*	Ex1	c.535C>T	p. (Gln179*)	Pathogenic (PVS1, PM2, PP4)	−3.3	IIIB	[21] PMID: 17041877
ARG73 (EM39)	14.6	FH	M	*EXT1*	Ex1	c.706dup	p. (Leu236Profs*4)	Pathogenic (PVS1, PMM2, PP4)	−1.9	IIIB	This study
ARG75 (EM43)	8.1	S	F	*EXT1*	Ex1	c.249del	p. (Gln84Argfs*52)	Pathogenic (PVS1, PM2, PP4, PP5)	−0.2	IB	[47] PMID: 9150727
ARG76 (EM44)	15.5	S	M	*EXT1*	Ex1	c.952G>T	p. (Glu318*)	Pathogenic (PVS1, PM2, PP4)	−0.4	IIA	This study
ARG77 (EM41)	37.2	FH	M	*EXT1*	Ex6	c.1469del	p. (Leu490Argfs*9)	Pathogenic (PVS1, PM2, PP4)	−1.9	IIIB	[50] PMID: 7550340 [4] PMID: 23439489 [35] PMID: 29126381 [34] PMID: 30806661 [46] PMID: 33632255
ARG78 (EM45)	9.7	S	M	*EXT1*	Ex10	c.2029C>T	p. (Gln677*)	Pathogenic (PVS1, PM2, PP4, PP5)	−0.1 neurological delay	IIA	[36] PMID: 16283885
ARG79 (EM46)	9.9	FH	M	*EXT1*	Ex10	c.1913_1916dup	p. (Leu642Glnfs*13)	Pathogenic (PVS1, PM2, PP4, PP5)	−0.7	IB	[21] PMID: 17041877
ARG80 (EM49)	21.0	S	F	*EXT1*	Ex1	c.288del	p. (Lys97Serfs*39)	Pathogenic (PVS1, PM2, PP4)	−3.0	IIIB	This study
ARG81 (EM50)	16.4	S	M	*EXT1*	Int4	c.1284+1G>T	p. ?	Pathogenic (PVS1, PM2, PP4)	−0.5	IIIB	[27] PMID: 16088908 [34] PMID: 30806661
ARG84 (EM54)	13.6	FH	M	*EXT1*	Ex3	c.1087G>T	p. (Gly363*)	Pathogenic (PVS1, PM2, PP4)	−0.8 neurological delay	IIIB	This study
ARG85 (EM55)	20.3	S	F	*EXT1*	Int3	c.1164+2T>A	p. ?	Pathogenic (PVS1, PM2, PP4)	−2.4	IIIA	This study
ARG86 (EM56)	14.6	FH	M	*EXT1*	Ex6	c.1469del	p. (Leu490Argfs*9)	Pathogenic (PVS1, PS4, PM2, PP4, PP5)	−0.1	IIIA	[50] PMID: 7550340 [4] PMID: 23439489 [35] PMID: 29126381 [34] PMID: 30806661
ARG52 (EM6)	16.5	FH	M	*EXT2*	Ex3	c.560T>G	p. (Leu187Arg)	VUS (PM2, PP3, PP4, BP1)	not included	not included	This study Hypochondroplasia *FGFR3* NM_000142.4: c.1620C>A p. (Asn540Lys)
ARG57 (EM10)	5.4	S	F	*EXT2*	Ex4	c.760del	p. (Leu254Serfs*16)	Pathogenic (PVS1, PM2, PP4, PP5)	0.0	IIA	[42] PMID: 29529714
ARG59 (EM16)	10.2	S	M	*EXT2*	Ex6	c.1016G>A	p. (Cys339Tyr)	VUS (PM2, PP3, PP4, BP1)	−1.2	IB	[33] PMID: 19839753 [45] PMID: 30334991 [34] PMID: 30806661
ARG63 (EM19)	8.0	FH	F	*EXT2*	Ex2	c.514C>T	p. (Gln172*)	Pathogenic (PVS1, PS4, PM2, PP4)	−0.1	IIA	[51] PMID: 8894688 [47] PMID: 29909963 [48] PMID: 32293802
ARG67 (EM27)	20.9	S	M	*EXT2*	Ex2	c.210del	p. (Arg70Serfs*42)	Pathogenic (PVS1, PM2, PP4)	−0.4	IIA	This study
ARG69 (EM29)	20.9	FH	F	*EXT2*	Ex3	c.560T>G	p. (Leu187Arg)	VUS (PM2, PP3, PP4, BP1)	−0.1	IIA	This study
ARG70 (EM33)	12.6	S	F	*EXT2*	Int7	c.1173+1G>A	p. ?	Pathogenic (PVS1, PM2, PP4)	0.8	IIIB	[23] PMID: 8894688
ARG72 (EM35)	18.8	FH	M	*EXT2*	Ex8	c.1234C>T	p. (Glu412*)	Pathogenic (PVS1, PM2, PP4)	−1.4	IB	[32] PMID: 10480354
ARG74 (EM42)	14.5	S	M	*EXT2*	Ex2	c.429C>G	p. (Tyr143*)	PVS1,	0.7	IIA	This study
ARG82 (EM51)	2.2	S	M	*EXT2*	Ex2	c.423del	p. (Tyr142Thrfs*128)	Pathogenic (PVS1, PM2, PP4)	0.9	IB	This study
ARG87 (EM48)	9.1	FH	F	*EXT2*	Ex8	c.1201C>T	p. (Gln401*)	Pathogenic (PVS1, PM2, PP4)	−1.9	not included	[46] PMID: 9326317 Turner syndrome

Transcripts—*EXT1*: NM:000127.3; *EXT2*: NM:000401.2. * According to classification by Pedrini et al.; 2011. FH—Family history. S—Sporadic. (p.?) a change in the sequence of bases in a DNA molecule, but do not result in a change in the amino acid sequence of a protein.

**Table 2:** *EXT1* and *EXT2* genotype-phenotype correlation in index patients. Univariate analysis (*n*:48).

Variable	*EXT1* (*n* = 26)	*EXT2* (*n* = 22)	*p* Value
Child (*n*) Adults	15 11	14 8	0.68
Gender (*n*) Female Male	12 14	8 12	0.49
Family history of Osteochondromatosis (*n*) Yes No	15 11	16 6	0.28
Age–year x (SD)	17.4 (11.6)	14.8 (10.7)	0.43
Age first symptom x (SD)	3.7 (5.6)	3.4 (3.9)	0.84
Clinical class (*n* =46) I II–III	5 21	10 10	0.03 *
Height z score x (SD)	−1.19 (1.38)	−0.16 (0.77)	0.003 **
Pain–yes (*n*)	17	11	0.09 *
Number of osteochondromas (*n*) ≥10	17/24	8/17	0.12
Surgery required (*n*)	17	10	0.17
Number of surgeries per child median (IQR)	2.0 (2–4)	2.5 (1–3)	0.25

* Fisher’s Exact test, ** two sample *t*-test.

## Data Availability

The datasets generated during and/or analyzed during the current study are not publicly available, but are available from the corresponding author on reasonable request.
